# Mediating role of avoidance of trauma disclosure and social disapproval in ICD-11 post-traumatic stress disorder and complex post-traumatic stress disorder: cross-sectional study in a Lithuanian clinical sample

**DOI:** 10.1192/bjo.2021.1055

**Published:** 2021-11-19

**Authors:** Monika Kvedaraite, Odeta Gelezelyte, Thanos Karatzias, Neil P. Roberts, Evaldas Kazlauskas

**Affiliations:** Center for Psychotraumatology, Institute of Psychology, Vilnius University, Lithuania; Center for Psychotraumatology, Institute of Psychology, Vilnius University, Lithuania; School of Health & Social Care, Edinburgh Napier University, UK; and NHS Lothian, Rivers Centre for Traumatic Stress, Edinburgh, UK; National Centre for Mental Health (NCMH), Division of Psychological Medicine and Clinical Neurosciences, Cardiff University School of Medicine, UK; and Directorate of Psychology and Psychological Therapies, Cardiff & Vale University Health Board, Cardiff, UK; Center for Psychotraumatology, Institute of Psychology, Vilnius University, Lithuania

**Keywords:** ICD-11, trauma disclosure, social disapproval, PTSD, complex PTSD

## Abstract

**Background:**

ICD-11 includes a new diagnosis of complex post-traumatic stress disorder (CPTSD), resulting predominantly from reoccurring or prolonged trauma. Previous studies showed that lack of social support is among the strongest predictors of PTSD, but social factors have been sparsely studied in the context of the ICD-11 definition of PTSD and CPTSD.

**Aims:**

To analyse the factor structure of the International Trauma Questionnaire (ITQ) in a Lithuanian clinical sample and to evaluate the mediating role of social and interpersonal factors in the relationship between trauma exposure and ICD-11 PTSD and CPTSD.

**Method:**

The sample comprised 280 adults from out-patient mental health centres (age, years: mean 39.48 (s.d. = 13.35); 77.5% female). Trauma-related stress symptoms were measured with the ITQ. Social disapproval was measured with the Social Acknowledgment Questionnaire (SAQ) and trauma disclosure using the Disclosure of Trauma Questionnaire (DTQ).

**Results:**

ICD-11 PTSD and CPTSD prevalence among the participants in this study was 13.9% and 10.0% respectively. Results indicated that avoidance of trauma disclosure mediated the relationship between trauma exposure and PTSD as well as CPTSD, whereas social disapproval mediated only the relationship between trauma exposure and CPTSD.

**Conclusions:**

The findings suggest that disclosure of traumatic experiences and support from closest friends and family members might mitigate the effects of traumatic experiences, potentially reducing the risk of developing CPTSD.

A new diagnosis of complex post-traumatic stress disorder (CPTSD) along with post-traumatic stress disorder (PTSD) was included in the recently published ICD-11.^1^ CPTSD diagnosis comprises the three PTSD symptom clusters: (a) re-experiencing, (b) avoidance and (c) sense of threat; as well as three symptom clusters of disturbances in self-organisation (DSO): (a) affect dysregulation, (b) negative self-concept and (c) disturbances in relationships.

PTSD and CPTSD are both very disabling disorders and often can have a late onset or chronic course. Identifying underlying risk and protective factors is therefore particularly important for the mitigation of risk and development of care for trauma survivors. Previous studies, using DSM-5 or ICD-10 definitions of PTSD, identified various peri-trauma or post-trauma risk factors of post-traumatic stress, but it has been consistently reported that inadequate social support is a strong factor contributing to the development and maintenance of PTSD;^2,3^ in particular, social approval from close friends and family^4^ and emotional social support^5^ have a substantial mediating (protective) effect between lifetime trauma exposure and PTSD. Even if it is generally accepted that lack of social support is one of the prominent risk factors for the onset and continuation of PTSD symptoms,^2,6^ further research is needed to explore the underlying risk factors for CPTSD. In particular, the role of social factors has been sparsely studied in the context of the new ICD-11 definitions of PTSD and CPTSD. Given the nature of the symptom profile of CPTSD, which includes exaggerated negative beliefs and disturbed relationships with others, it is plausible that social support is associated more strongly with CPTSD symptoms than PTSD symptoms.^7^

Social support is a broad and multidimensional concept, but research shows that disclosure of trauma^8^ and social acknowledgement^9^ are among the most relevant social support factors following traumatic experiences. Disclosing trauma and trauma-related problems following the traumatic experiences has been shown to have positive therapeutic effects; in contrast, the lack of disclosure can predict stronger ICD-10 PTSD reactions.^10^ Furthermore, traumatic events that are more commonly associated with CPTSD reactions, such as sexual and childhood trauma, were associated with greater difficulty in disclosing,^11^ alongside increased experience of negative self-referential emotions such as shame.^12^ However, results of the trauma disclosure effects are inconsistent, with some studies showing negative effects of disclosing.^13^ These results can be partially explained by negative social reactions towards trauma survivors who choose to disclose their experiences. Therefore it is important to study the effects of disclosure of trauma in tandem with social acknowledgement and approval. To our knowledge, disclosure of trauma has not yet been studied in the context of the ICD-11 definition of CPTSD, and therefore links between social acknowledgement and CPTSD are unknown. One study relevant to the exploration of the effects of social factors on CPTSD shows that those with a higher risk for CPTSD exhibit lower levels of perceived social support, even when compared with a PTSD group.^7^ Taking into account these studies and updates in ICD-11, we find it important to further explore the lack of social acknowledgement and avoidance of disclosure of the trauma event as relevant risk factors for the onset and maintenance of CPTSD. A more comprehensive understanding of the role of social risk factors in traumatic stress would also be in line with a social–interpersonal framework model of PTSD,^14^ which states that the social and interpersonal context (social affects, interpersonal relationships, culture and society) is an important factor not only for the onset of PTSD but also for resilience and positive adaptation following traumatic experiences.

Furthermore, the recent CPTSD diagnosis has created the need for new methods of assessment.^15^ The symptom structure of ICD-11 PTSD and CPTSD has been validated across multiple samples, and a new instrument was developed to specifically measure ICD-11 PTSD and DSO symptoms – the International Trauma Questionnaire (ITQ).^16^ Previous studies in Lithuania evaluated the test version of the 22-item ITQ and found that the factor structure was in line with the ICD-11 PTSD and CPTSD formulation.^17^ Since then, a shorter 12-item ITQ version has been developed after validation in various samples.^16^ However, the 12-item ITQ factor structure has not been tested in a Lithuanian sample yet, and researchers and clinicians in Lithuania do not have reliable measures to screen for PTSD and CPTSD symptoms. This shortage of reliable measures may be one of the reasons for poor recognition of trauma-related disorders in Lithuanian mental health services.^18^

This study aimed to (a) evaluate the structural validity of the ITQ in a Lithuanian sample of mental health service patients and (b) assess the role of social–interpersonal factors (i.e. avoidance of trauma disclosure and lack of social approval from friends and family) in PTSD and CPTSD. We hypothesised that avoidance of trauma disclosure and lack of social approval from friends and family would mediate PTSD and CPTSD following exposure to trauma.

## Method

### Participants and procedure

A dataset for analysis in this study was obtained from a larger research project on the ICD-11 stress-related disorders conducted by the Vilnius Center for Psychotraumatology, Lithuania. A secondary analysis of the previously unpublished data was conducted. Results of the larger research project have been published previously.^17^ The study was approved by the Institutional Psychological Research Ethics Committee at Vilnius University (2016/04/05 Nr.8). Participants were recruited by 20 psychologists in multiple cities across Lithuania. The settings for data collection included private clinical psychologists’ practice, primary mental health centres, hospitals and out-patient mental health clinics. All participants provided written informed consent before data collection.

In total, 348 adults provided written informed consent for participation in the study, a response rate of 81.1%. The data of 68 participants were not included in analysis because they reported no previous trauma experiences (*n* = 29) or did not complete the PTSD and CPTSD assessments (*n* = 39). The final sample comprised 280 participants: 217 (77.5%) female, mean age 39.48 years (s.d. = 13.35), age range 18–84 years. The majority of participants (79.3%, *n* = 222) lived in an urban area, around two-thirds (63.9%, *n* = 179) were employed and around one-third (37.9%, *n* = 106) had a university degree. More information on sociodemographic characteristics of the PTSD, CPTSD and no-PTSD groups is presented in Table 1. We found no significant differences regarding the sociodemographic characteristics among these groups.

**Table 1:** Sociodemographic characteristics of the study sample (*n* = 280)

Variable	No PTSD (*n* = 213) *n (%)*	PTSD (*n* = 39) *n (%)*	CPTSD (*n* = 28) *n* (%)	Significance statistics
Gender				
Male	43 (20.2)	12 (30.8)	8 (28.6)	χ^2^(2) = 2.77
Female	170 (79.8)	27 (69.2)	20 (71.4)	
Age				
Mean (s.d.)	39.52 (13.77)	41.75 (12.15)	36.32 (11.53)	*F*(2) = 1.31
Range	18–84	18–69	19–58	
Relationship status				
In relationship	133 (63.3)	23 (60.5)	12 (42.9)	χ^2^(2) = 4.35
Single	77 (36.7)	15 (39.5)	16 (57.1)	
Children				
Yes	135 (63.4)	29 (74.4)	18 (64.3)	χ^2^(2) = 1.75
No	78 (36.6)	10 (25.6)	10 (35.7)	
Residence				
Urban	171 (80.7)	32 (82.1)	19 (67.9)	χ^2^(2) = 2.67
Rural	41 (19.3)	7 (17.9)	9 (32.1)	
Education				
University degree	86 (40.6)	13 (33.3)	7 (25.0)	χ^2^(4) = 8.22
Secondary non university education or some college	68 (32.0)	19 (48.8)	9 (32.1)	
High/secondary school or lower	58 (27.4)	7 (17.9)	12 (42.9)	
Employment				
Employed	142 (68.3)	21 (53.8)	16 (57.1)	χ^2^(2) = 3.87
Not employed	66 (31.7)	18 (46.2)	12 (42.9)	
Income				
Average or higher	80 (48.5)	14 (46.7)	12 (52.2)	χ^2^(2) = 0.16
Lower than average	85 (51.5)	16 (53.3)	11 (47.8)	

PTSD, post-traumatic stress disorder; CPTSD, complex PTSD.

### Measures

The Life Events Checklist (LEC) was used to measure trauma exposure in the sample.^19^ We used the revised version, which lists 18 different traumatic experiences, with two additional items added to the standard version measuring physical abuse in childhood and sexual abuse in childhood.^20,21^ Participants were asked to report whether they have experienced, witnessed, learned about or never have been exposed to the listed experiences. Experiencing or witnessing a potentially traumatic event was regarded as exposure to trauma. Previous studies showed adequate stability and association with PTSD symptoms.^22^ We used the sum of traumatic experiences to estimate cumulative trauma exposure in our sample.

The International Trauma Questionnaire (ITQ)^16^ was used to assess ICD-11 PTSD and CPTSD. The ITQ comprises 12 symptom items that assess 6 symptom clusters in total (two items per cluster). Three of the clusters are for PTSD: re-experiencing, avoidance and sense of current threat; and three are for DSO: affective dysregulation, negative self-concept and disturbances in relationships. Participants indicated the intensity of the listed PTSD symptoms over the previous month. For the DSO assessment, participants were asked to indicate how they typically feel, think about themselves and relate to others. After PTSD and DSO symptom items, the questionnaire lists functional impairment items associated with problems in: relationships and social life, work or ability to work, and any other important part of life. All items were rated from 0 (not at all) to 4 (extremely). Endorsement of a symptom or functional impairment is defined as a score ≥2.^16^ As the algorithm of the ITQ indicates, the diagnosis of PTSD requires the endorsement of one of two symptoms from each PTSD cluster, plus the endorsement of functional impairment related to these symptoms. CPTSD is diagnosed if a person meets criteria for PTSD and all three DSO symptom clusters are endorsed, along with at least one DSO-related functional impairment item.^16^ In the current study, Cronbach's alpha for the total ITQ score was 0.88, for the PTSD symptoms α = 0.86 and for the DSO symptoms α = 0.85.

Avoidance of trauma disclosure was measured using the 12-item Disclosure of Trauma Questionnaire (DTQ-12).^8^ For the purposes of this study, we used the Reluctance to Talk subscale, which consists of four items specifically associated with avoidance of trauma disclosure. Each item was rated on a Likert scale ranging from 0 (not at all) to 5 (completely), evaluating how much participants are willing to disclose the most troubling traumatic experience. The DTQ-12 score was computed by summing all responses: higher scores indicated a stronger reluctance to disclose traumatic experiences. Previous studies have shown good reliability for the Lithuanian version of the DTQ-12.^23,24^ Cronbach's α for the Reluctance to Talk subscale in this study was 0.77.

Social acknowledgement from family and friends was measured using items extracted from the Social Acknowledgment Questionnaire (SAQ).^9^ In this study, we used only the five-item Family and Friends Disapproval subscale, which is related to social acknowledgement from family and friends, to estimate participant's interaction with the closest social context. Participants rated on a Likert scale ranging from 0 (completely disagree) to 3 (completely agree) how much their friends or family members support or understand them and their experiences concerning the most troubling traumatic experience referred to in the ITQ. The total score was computed by summing all responses: higher scores indicated stronger social disapproval from friends and family. Cronbach's α in this study was 0.58. The low value of Cronbach's α for the social disapproval measure could in part be explained by the low number of items. Nevertheless, an alpha ranging from 0.5 to 0.7 still shows moderate reliability and is acceptable in research.^25^

### Data analysis

**Fig. 1:**
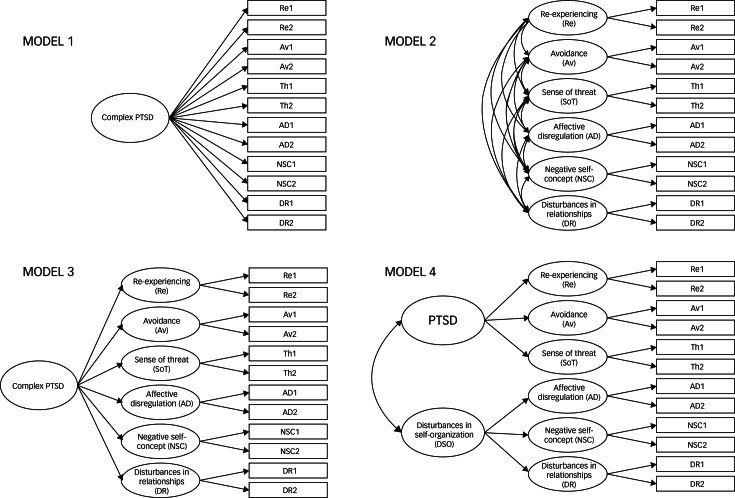
ICD-11 post-traumatic stress disorder (PTSD) and complex PTSD (CPTSD) confirmatory factor analysis models.

To test the factor structure and the validity of the ITQ scale, we conducted confirmatory factor analysis (CFA). In our analysis we tested four alternative CFA models, which were described in a previous study^26^ and are presented in Fig. 1. These CFA models were estimated using the Robust Maximum Likelihood (MLR) estimator method.

The mediating role of social factors on the relationship between traumatic exposure and PTSD and CPTSD was tested by applying the Structural Equation Modelling (SEM) approach. We included PTSD and CPTSD diagnosis as binary variables in the SEM model, computed using the ITQ diagnostic algorithm. When testing the models, we used the weighted least square mean and variance adjusted (WLSMV) estimation method. We tested both direct and indirect (or mediated) links between the study variables. Indirect effects were tested using a bootstrap estimation approach with 5000 samples.^27^ Two alternative SEM models were tested. The first SEM model did not include any control variables, the second SEM model was estimated after controlling for gender, age, education and relationship status effects on social factors, as these factors have been shown to independently associate with either PTSD or CPTSD.^28^ Model fit for the CFA and SEM models was assessed using the root mean square error of approximation (RMSEA), the chi-square test, the standardised root mean square residual (SRMR) indices, the comparative fit index (CFI), and the Tucker–Lewis index (TLI). CFI and TLI values >0.90 and RMSEA and SRMR values ≤0.08 indicate acceptable model fit.^28^

IBM SPSS Statistics 25 (for Windows) was used for descriptive statistics analyses. Mplus 8.2 (for Windows) was used to conduct the CFA and SEM analysis.

## Results

### Trauma exposure

Participants reported experiencing an average of 5.77 (s.d. = 2.97) different lifetime traumatic experiences, with each participant reporting between 1 and 16 types (from those listed in the LEC). Exposure to only 1 trauma type was reported by 3.9% (*n* = 11), 2–3 trauma types were reported by 21.1% (*n* = 59), 4–6 types were experienced by 35.7% (*n* = 100), 7–9 types were reported by 27.9% (*n* = 78) and ≥10 types were reported by 11.4% (*n* = 32) of participants. The most prevalent trauma experiences were sudden accidental death of a loved one (72.1%), severe human suffering (67.9%), transportation accidents (60.7%) and physical assault (58.9%).

In total, 39 (13.9%) participants met the probable diagnostic criteria for PTSD and 28 (10.0%) for CPTSD. Significant differences between the non-PTSD, PTSD and CPTSD groups’ exposure to different trauma experiences were found. The CPTSD group reported experiencing more lifetime types of trauma (7.75; s.d. = 2.77) than the non-PTSD group (5.40; s.d. = 2.95) (*F*(2) = 9,1; *P <* 0.001). The CPTSD group reported experiencing more childhood physical abuse (χ^2^(2) = 15.83; *P <* 0.001) than the PTSD and non-PTSD groups, whereas the PTSD group reported experiencing more physical assault (χ^2^ (2) = 8.19; *P =* 0.017) than the non-PTSD and CPTSD groups. In addition, the CPTSD and PTSD groups reported experiencing more assault with a weapon (χ^2^(2) = 11.20; *P =* 0.004) and unspecified unwanted or uncomfortable sexual experience (χ^2^(2) = 8.11; *P =* 0.017) than the non-PTSD group.

### Analysis of PTSD and CPTSD symptom structure

Model fit statistics for all the tested CFA models are presented in Table 2. Model 4 had a non-significant chi-square result and demonstrated acceptable fit based on the CFI, TLI, SRMR and RMSEA values. Thus, the second-order two-factor model (Model 4) has been chosen as the best fitting model in line with CPTSD theoretical conceptualisation. Factor loadings and all correlations among the latent factors were significant (*P* < 0.001) (Table 3). Factor loadings ranged between 0.52 and 0.95; the correlation between latent factors ranged between 0.72 and 1.00. Owing to the negative covariance matrix error, the correlation between DSO and affective dysregulation was constrained at 1. The correlation between DSO and PTSD factors was 0.60.

**Table 2:** Model fit statistics for the tested confirmatory factor analysis models

Model	RMSEA (90% CI)	SRMR	CFI	TLI	χ^2^ (d.f.), *P*
Model 1	0.230 (0.216–0.243)	0.121	0.560	0.462	851.77 (54), <0.001
Model 2	0.055 (0.034–0.074)	0.111	0.976	0.961	73.58 (40), <0.001
Model 3	0.093 (0.077–0.109)	0.083	0.918	0.887	163.65 (48), <0.001
**Model 4**	**0.029** (**0.000–0.051)**	**0**.**042**	**0**.**992**	**0**.**989**	**58.09 (47), 0.129**

RMSEA, root mean square error of approximation; SRMR, standardised root mean square residual; CFI, comparative fit index; TLI, Tucker–Lewis index.

Bold denotes the best fitting model.

**Table 3:** Standardised factor loadings and standard errors for the second-order two-factor model (Model 4)^a^

Items	Re-experiencing	Avoidance	Sense of current threat	Affect dysregulation	Negative self-concept	Disturbances in relationships
Re1	0.73 (0.04)					
Re2	0.86 (0.04)					
Av1		0.83 (0.03)				
Av2		0.93 (0.03)				
SoT1			0.75 (0.03)			
SoT2			0.90 (0.03)			
AD 1				0.58 (0.05)		
AD 2				0.52 (0.05)		
NSC 1					0.94 (0.02)	
NSC 2					0.95 (0.02)	
DR 1						0.89 (0.03)
DR 2						0.78 (0.03)
First-order factors	PTSD			Disturbances in self-organisation		
Re	0.77 (0.04)					
Av	0.72 (0.04)					
SoT	0.97 (0.04)					
AD				1.00 (0.00)		
NSC				0.77 (0.04)		
DR				0.77 (0.04)		

Re, re-experiencing; AV, avoidance; SoT, sense of current threat; AD, affect dysregulation; NSC, negative self-concept; DR, disturbances in relationships; PTSD, post-traumatic stress disorder.

a. All factor loadings are statistically significant (*P* < 0.001).

### Social factors, PTSD and CPTSD

We found that the CPTSD group experienced stronger social disapproval from family and friends than the PTSD and non-PTSD groups, and both the PTSD and CPTSD groups reported higher avoidance of trauma disclosure than the non-PTSD group (Table 4).

**Table 4:** Symptoms and social factors by group (*n* = 280)^a^

Variable	Non-PTSD group (*n* = 213) Mean (s.d.)	PTSD group (*n* = 39) Mean (s.d.)	CPTSD group (*n* = 28) Mean (s.d.)	Significance statistics	
				*F* (d.f. = 2)	*P*
CPTSD symptoms	12.26 (7.93)^b,c^	23.85 (5.12)^a,c^	32.21 (5.93)^a,b^	115.28	<0.001
PTSD symptoms	5.78 (4.65)^b,c^	15.74 (3.97)^a^	16.25 (3.59)^a^	132.30	<0.001
Re-experiencing	1.50 (1.78)^b,c^	4.87 (1.88)^a^	4.82 (2.09)^a^	85.50	<0.001
Avoidance	2.21 (2.38)^b,c^	5.54 (1.65)^a^	6.07 (1.30)^a^	66.22	<0.001
Sense of threat	2.07 (2.09)^b,c^	5.33 (1.58)^a^	5.36 (1.54)^a^	70.07	<0.001
DSO symptoms	6.48 (5.05)^b,c^	8.10 (2.98)^a,c^	15.96 (3.74)^a,b^	50.56	<0.001
Affect dysregulation	2.53 (1.60)^b,c^	3.36 (1.29)^a,c^	4.75 (1.71)^a,b^	26.93	<0.001
Negative self-concept	1.69 (2.23)^c^	1.90 (2.00)^c^	5.86 (1.98)^a,b^	45.53	<0.001
Disturbed relationships	2.26 (2.17)^c^	2.85 (1.99)^c^	5.36 (1.39)^a,b^	27.62	<0.001
Social disapproval from family/friends	5.91 (2.85)^c^	6.11 (2.70)^c^	8.80 (2.63)^a,b^	11.83	<0.001
Avoidance of trauma disclosure	6.62 (4.47)^b,c^	9.68 (3.65)^a^	12.07 (4.45)^a^	23.85	<0.001

PTSD, post-traumatic stress disorder; CPTSD, complex post-traumatic stress disorder; DSO, disturbances in self-organisation.

a. Superscripts denote significant differences at *P* < 0.05 for the respective groups: ^a^, non-PTSD; ^b^, PTSD; ^c^, CPTSD.

**Fig. 2:**
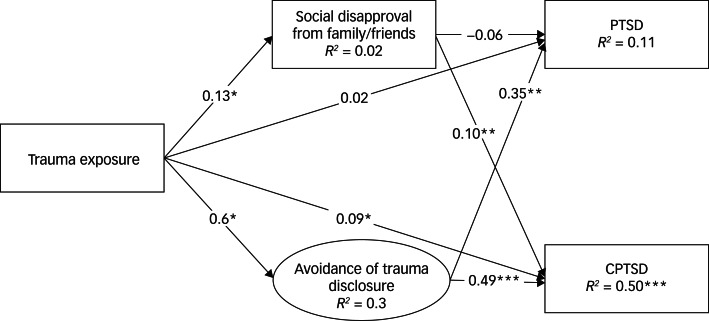
Model of post-traumatic stress disorder (PTSD) and complex PTSD (CPTSD) symptoms through social disapproval from friends and family and avoidance of trauma disclosure. *P < 0.05, **P < 0.01, ***P < 0.001.

The mediating role of social factors on PTSD and CPTSD when exposed to traumatic events was tested by applying the SEM approach. The final SEM model of the role of social factors on PTSD and CPTSD is shown in Fig. 2. In this model, trauma exposure predicted avoidance of trauma disclosure and social disapproval from friends and family for PTSD and CPTSD. PTSD and CPTSD were also predicted by social disapproval from friends and family and avoidance of trauma disclosure. The model explained significant levels of variance in all variables (*P* < 0.05) except for the effects of trauma exposure and social disapproval from friends and family on PTSD. We therefore included the estimation of the indirect effects of accumulative exposure on traumatic events through social disapproval from friends and family and avoidance of trauma disclosure to CPTSD. The estimation of the model yielded good model fit (χ^2^(14) = 19.91, *P* = 0.133, CFI/TLI = 0.972/0.944, RMSEA 90% CI 0.039 (0.000–0.076), SRMR = 0.051). We also tested an alternative model controlling for gender, age and relationship status effects on avoidance of trauma disclosure and social disapproval from friends and family. However, an alternative model yielded unacceptable model fit (χ^2^(26) = 51.32, *P* = 0.002, CFI/TLI = 0.890/0.810, RMSEA 90% CI 0.060 (0.036–0.084), SRMR = 0.036). As the final model, we therefore chose the one without control variables.

Trauma exposure was significantly directly associated with CPTSD, but not with PTSD when social disapproval from friends and family and avoidance of trauma disclosure were included as mediators (Fig. 2). We also found that both social disapproval from friends and family and avoidance of trauma disclosure were significantly directly related to CPTSD symptoms. However, only avoidance of trauma disclosure was directly related to PTSD symptoms. Additionally, we found that the indirect link between trauma exposure and CPTSD symptoms through avoidance of trauma disclosure (IND = 0.08 (0.02–0.15)) was significant but weak. All other indirect links were found to be non-significant.

## Discussion

This is one of the first studies analysing the role of social–interpersonal factors on ICD-11 PTSD and CPTSD in a clinical sample revealing the different effects of avoidance of disclosure and social disapproval on the two disorders. Our results show that social–interpersonal factors are important mediators for both PTSD and CPTSD.

In our study, avoidance of trauma disclosure significantly mediated the relationship between traumatic exposure and the risk for ICD-11 PTSD and CPTSD. These results are in line with previous research which shows that, although survivors with PTSD often avoid being open about their traumatic experiences,^29^ disclosure is related to well-being and lower levels of PTSD symptoms.^14^ Furthermore, our results suggest that disclosure of trauma is no less important in the case of CPTSD – a diagnostically new condition.

More important, our results showed that social disapproval from family and friends associated with trauma exposure was a significant mediator for CPTSD but not for PTSD. Simon et al^7^ also found significant associations between perceived social support and the disturbances in self-organisation (DSO) symptom clusters in CPTSD, whereas relationships between social support and symptom clusters in PTSD were non-significant. These findings indicate that the lack of support from friends and family contributes to more adverse trajectories of psychopathology after traumatic experiences, which might result in complex post-traumatic stress symptoms. However, these results should be interpreted carefully, as the instrument used to measure social support showed moderate reliability. Furthermore, this is a cross-sectional study and it is equally possible that PTSD and DSO symptoms influence perceptions of social support and willingness to disclose. Moreover, the differences we found between PTSD and CPTSD in relation to disapproval from friends and family might be explained by additional DSO symptoms that CPTSD encompasses. PTSD can be perceived more as a conditioned fear response, whereas CPTSD represents a more complex effect of trauma resulting in disturbances of emotion regulation, self-identity and relationships with others.^15^

As this was the first study using the final version of the ITQ in a Lithuanian clinical sample, we also aimed to evaluate the factor structure of the ITQ in a Lithuanian sample of mental health service users. In line with previous studies,^17,30–32^ we found that the two-factor second-order model where a second-order PTSD factor accounts for the covariation between the re-experiencing, avoidance and sense of current threat factors and a second-order DSO factor accounts for the covariation between the affect dysregulation, negative self-concept and disturbances in relationships factors had a good fit. This model was consistent with ICD-11 diagnostic criteria for PTSD and CPTSD. The ITQ factor structure and reliability analysis also indicate that this measure can be used in Lithuania in clinical practice for PTSD and CPTSD screening.

The prevalence of PTSD and CPTSD among the mental health patients in this study was 23.9%. In total, 13.9% of participants met the criteria for PTSD and 10.0% for CPTSD. Other studies with treatment-seeking samples (who experienced sexual abuse, childhood abuse or were referred to the psychologist for other reasons) reported a similar or even higher prevalence of trauma-related disorders.^33,34^ However, the rates of PTSD and CPTSD prevalence in general population studies are lower.^28,31,35^ Thus, these findings indicate that PTSD and CPTSD symptoms are highly prevalent among mental health patients in Lithuania and that recognising stress-related symptoms and their risk factors in a clinical setting is very important.

### Limitations and future research

The study has several limitations. First, this is a cross-sectional study, which limits causal inferences and makes the identified associations more challenging to interpret and more susceptible to biases. Future studies should explore social factors in relation to PTSD and CPTSD in a longitudinal study. Further studies could also explore how social factors, such as disclosure of trauma or acknowledgement from family members, contribute to recovery following traumatic experiences. Another limitation of this study is the low, although still of moderate reliability, Cronbach's α of the Social Acknowledgment Questionnaire. The results related to social acknowledgement should be interpreted carefully, and other instruments should be considered for measuring social acknowledgement. In the current study, we also did not collect data regarding possible comorbidities of various disorders related to PTSD and CPTSD, such as borderline personality disorder, depression and anxiety. Further studies could benefit from including this data as it might help to better identify risk factors that are specifically related to PTSD or CPTSD. Furthermore, we used self-report measures to evaluate PTSD and CPTSD. Although the ITQ is a widely used instrument for ICD-11 post-traumatic stress disorders, diagnostic interviews such as the International Trauma Interview might provide more extensive information on which to make diagnostic decisions.^36^

### Clinical implications

Our findings suggesting that supporting disclosure of traumatic experiences in an empathic way might mitigate the effects of traumatic experiences require further investigation, but are nonetheless important for clinicians who are providing treatment for trauma survivors. The inclusion of family members, partners or friends in the treatment process could foster therapeutic effects in the treatment of CPTSD. Furthermore, providing education to the general population on the effects of traumatic experiences might help family members and friends interact in a more understanding and respectful way with close ones who have experienced traumatic experiences.

## Data Availability

The raw data supporting the conclusions of this article are available from the corresponding author on request.
